# Monocyte distribution width (MDW) as a useful indicator for early screening of sepsis and discriminating false positive blood cultures

**DOI:** 10.1371/journal.pone.0279374

**Published:** 2022-12-20

**Authors:** Sung Jin Jo, Sei Won Kim, Jung-Hyun Choi, Seoung Pill Choi, Jehoon Lee, Jihyang Lim

**Affiliations:** 1 Department of Laboratory Medicine, Eunpyeong St. Mary’s Hospital, College of Medicine, The Catholic University of Korea, Seoul, Korea; 2 Division of Pulmonary, Critical Care and Sleep Medicine, Department of Internal Medicine, Eunpyeong St. Mary’s Hospital, College of Medicine, The Catholic University of Korea, Seoul, Korea; 3 Division of Infectious Disease, Department of Internal Medicine, Eunpyeong St. Mary’s Hospital, College of Medicine, The Catholic University of Korea, Seoul, Korea; 4 Department of Emergency Medicine, Eunpyeong St. Mary’s Hospital, College of Medicine, The Catholic University of Korea, Seoul, Korea; The Ohio State University, UNITED STATES

## Abstract

**Background:**

Severe sepsis and septic shock are the leading cause of in-hospital death. As sepsis progresses, expression and activity of endogenous mediators of inflammation change. Early detection of biomarkers can play a role in sepsis screening and in improvement of patient outcomes. Recent studies suggest that increase in monocyte volume may be helpful in early detection of sepsis. Therefore, we evaluated the utility of monocyte distribution width (MDW) for the early assessment of sepsis compared with the blood culture and other inflammatory biomarkers.

**Methods:**

Medical records of 1,404 patients (aged ≥19 years) who were admitted to the emergency department owing to clinically suspected infectious disease and requested blood cultures from Oct 2019 to Jan 2021 were reviewed. The patients were grouped based on Sepsis-3 criteria. They had undergone other laboratory tests to evaluate their clinical status. MDW was analyzed using DxH900 hematology analyzer (Beckman Coulter, Brea, California, USA). To determine the diagnostic performance of MDW, C-reactive protein (CRP), and procalcitonin (PCT) for sepsis, the area under the curve (AUC) of receiver operating characteristics curves and their sensitivity and specificity were measured.

**Results:**

Among 1,404 patients, 520 patients were designated the sepsis group based on Sepsis-3 criteria. In the sepsis group, MDW value was 24.1 (median, IQR 21.6–28.1); AUC values for MDW, CRP, and PCT were 0.67 (95% CI, 0.64–0.69), 0.66 (95% CI, 0.63–0.68), and 0.75 (95% CI, 0.72–0.77), respectively. For diagnosis of the sepsis, the cut-off value of MDW was 21.7 (sensitivity 74% and specificity 54%). Measured values of MDW were higher for the blood culture positive group than that of the blood culture contamination group (P<0.001, 95% CI, -5.9 to -3.0) or blood culture negative group (P<0.001, 95% CI = -5.8 to -4.2).

**Conclusions:**

MDW is a new hematological parameter that is simultaneously calculated during complete blood cell counting by Beckman Coulter hematology analyzer. MDW is expected to serve as a useful indicator for early screening of sepsis in conjunction with CRP and PCT. MDW is especially useful for sepsis assessment in patients with a suspected infection. MDW can also assist in discriminating false positive blood cultures.

## Introduction

Complete blood count (CBC) is one of the earliest tests that can be performed in clinical practice for patients suspected of having a bacterial infection. While the level of white blood cells may increase or decrease in patients with sepsis, this response is nonspecific [1]. C-reactive protein (CRP) and procalcitonin (PCT) are biomarkers used in the basic laboratory tests for the diagnosis of sepsis [2,3]. However, these tests take a longer period of time than CBC, and it is possible that the clinician may not prescribe the test at the time of patients’ initial visit to the hospital. On the contrary, CBC is the simplest clinical test, where activation and morphological changes of neutrophils and monocytes in response to infection can be observed. Thrombocytopenia is also frequently observed as a response to sepsis [4]. As the CBC is a readily-performed, basic test, studies have investigated the use of the various parameters that are measured as part of the CBC with an automatic device; these include cell size, mean volume, and heterogeneity of each type of blood cells, as adjunct markers for the detection of sepsis or acute infection [5–12]. Elliott et al. reported that the monocytes distribution width (MDW), a hematologic parameter measured as part of the routine differential CBC test, has improved the detection of sepsis and that MDW outperformed the change in volumetric markers in the detection of sepsis [13]. Subsequent studies verified the utility of MDW in the diagnosis of early sepsis [14,15] and recent studies have compared the MDW in combination with the white blood cell (WBC) count against such biomarkers as the CRP or PCT to show that the ability of MDW to diagnose sepsis was either similar or better [16,17]. Additionally, recent studies have also investigated the clinical utility of increase in MDW for the detection of COVID-19 infection [18–20].

Monocytes are activated in response to infection, and they are divided into subsets depending on the chemokine receptor expression; this activation leads to an increase in MDW [4,21,22]. As MDW increases in response to bacterial infection in blood, we hypothesized that it is likely that MDW is related to the blood culture results. Although blood culture test is the gold standard in diagnosing bacterial infections in blood, low sensitivity and false positivity are still valid concerns [23,24].

Circulating WBCs in ethylene diamine tetra acetic acid (EDTA) tube were tested by hematology analyzer device that analyses blood cell volume, conductivity, and light scatter (VCS technology), which thus allows simultaneous measurements of the size of monocytes and MDW as part of the CBC investigation [25]. The present study was conducted to identify those factors that increase MDW, based on the hypothesis that MDW measurements are related to blood culture results. In this study, MDW as an early screening indicator of sepsis based on the Sepsis-3 criteria according to the third international consensus definitions for sepsis and septic shock [26] was assessed and the correlation of MDW value with the results of blood culture and other inflammatory biomarkers was also determined.

## Methods

The medical records of patients during the period from Oct 2019 to Jan 2021 were analyzed. The inclusion criteria for the study population were as follows: (i) male and female patients aged 19 years or above; (ii) those who were admitted to the emergency department owing to a suspected infectious disease; and (iii) those for whom the CBC, blood culture and other laboratory tests were performed within 12 hours in admission. This study was approved by the institutional review board at Eunpyeong St. Mary’s hospital (PC21RISE0021).

### Laboratory tests

CBC with MDW was measured using the DxH 900 hematology analyzer (Beckman Coulter, Brea, California, USA). MDW is simultaneously calculated as the standard deviation of a set of monocyte cell volume values during CBC. CRP and PCT were measured using the AU5800 chemistry analyzer (Beckman Coulter) and the UniCel® DxI 800 immunoassay analyzer (Beckman Coulter), respectively.

### Sepsis definition and disease classification

The patients’ medical records were reviewed and categorized according to Sepsis-3 criteria into the following three groups: controls, infection group, and sepsis group that classified according to Sequential Organ Failure Assessment [SOFA] criteria. To classify SOFA criteria, laboratory test results were analyzed including bacterial culture, PCR, antigen, antibody, CRP, PCT, and radiological tests performed within 12 hours of the emergency department admission. Although the test results within 12 hours were used to assess Sepsis-3 criteria, the clinicians used all the medical records available to evaluate correctly Sepsis-3 groups. Since this is a retrospective medical record study, all diagnoses, test results, and discharge record during the patient’s hospitalization period were reviewed. For the appropriate categorization of patients according to the criteria, two or more clinicians were involved in the decision-making process without knowing MDW value. The exclusion criteria were as follows: (i) CBC results obtained two or more hours after blood collection; (ii) patients that were admitted to the emergency department more than once to prevent duplication of data; (iii) patients that were discharged before adequate assessment; (iv) patients with an inadequately low monocyte count (less than 100/μL); and (v) patients with a history of hematological diseases. The patients were additionally grouped according to the infection area. Co-infection was defined as a case of infection two or more sites with blood culture-negative result.

### Blood culture

The blood culture was performed by BACTEC FX blood culture system (Becton Dickinson, Flanklin Lakes, NJ, USA). For bacterial identification, Bruker Biotyper system (Bruker Daltonics, Billerica, MA, USA) or the Microscan Walkaway 96 Plus system (Beckmann Coulter, Nyon, Switzerland) was used. One aerobic and one anaerobic bottles were regarded as one culture set. Blood samples were collected maximum of four culture sets depending on the patient’s clinical status. At least one bottle showed growth of the bacteria was determined as a positive blood culture except coagulase negative staphylococci (CNS), *Propionibacterium acnes*, *Corynebacterium* spp., and *Bacillus* spp. They were regarded as a blood culture contamination. However, two or more sets at the same collection time for CNS and viridans group were interpreted as blood culture positive [23].

### Statistical analysis

Statistical analysis and figure production was performed using the MedCalc software ver 20 (MedCalc Software, Ostend, Belgium) and GraphPad Prism ver 9.3.1 (GraphPad Software, San Diego, CA). The Kolmogorov–Smirnov test was used to assess the normality of the distribution. For between-group comparison, the Student *t* test or the Mann Whitney U test was used according to the groups. For the comparison of three or more groups, the ANOVA test or the Kruskall-Wallis test was used. The area under the curve (AUC) of MDW to predict sepsis was calculated using a Receiver Operating Characteristic (ROC) curve. To determine the optimal cut-off values of MDW, Youden’s index from ROC curve analysis was used. The statistical significance defined P-value of <0.05.

## Results

During the study period, a total of 1,615 patients were initially screened. Among them, 211 patients were excluded from this study because 90 patients had an inadequately low monocyte count; 65 patients were double participation; two patients were discharged before adequate assessment; and 54 patients had a hematological disease. Finally, total 1,404 patients were included in this study (Table 1).

**Table 1:** Characteristics of 1,404 patients and measured values of various parameters based on Sepsis-3 criteria.

Parameters		Three groups by Sepsis-3 criteria		
	Total (N = 1,404)	Control (N = 529)	Infection (N = 355)	Sepsis (N = 520)
Sex (N), male / female	710 / 694	261 / 268	214 / 141	235 / 285
Age (years), median (IQR)	71 (56–81)	66 (48–78)	66 (49–80)	75 (66–84)
WBC (x10^9^/L), median (IQR)	9.2 (6.8–12.7)	8.9 (6.8–11.8)	9.0 (6.7–12.4)	9.9 (6.9–13.8)
Neutrophil (x10^9^/L), median (IQR)	7.2 (4.7–10.4)	6.5 (4.5–9.7)	7.1 (4.5–9.8)	8.2 (5.1–11.9)
Monocyte (x10^9^/L), median (IQR)	0.65 (0.46–0.89)	0.66 (0.47–0.88)	0.68 (0.49–0.9)	0.62 (0.44–0.88)
**MDW, median (IQR)**	**22.6 (19.8**–**25.7)**	**21.0 (18.7**–**24.2)**	**22.8 (19.9–25.6)**	**24.1 (21.6–28.1)**
CRP (mg/dL), median (IQR)	6.4 (1.33–14.5)	2.7 (0.47–9.2)	7.6 (2.1–13.8)	10.6 (2.6–19.7)
PCT (ng/mL), median (IQR)	0.23 (0.06–1.49)	0.09 (0.03–0.32)	0.15 (0.05–0.53)	1.01 (0.18–6.9))

N: Number of patients; IQR: Inter quartile range; WBC: White blood cells; MDW: Monocyte distribution width; CRP: C-reactive protein; PCT: Procalcitonin.

**Fig 1:**
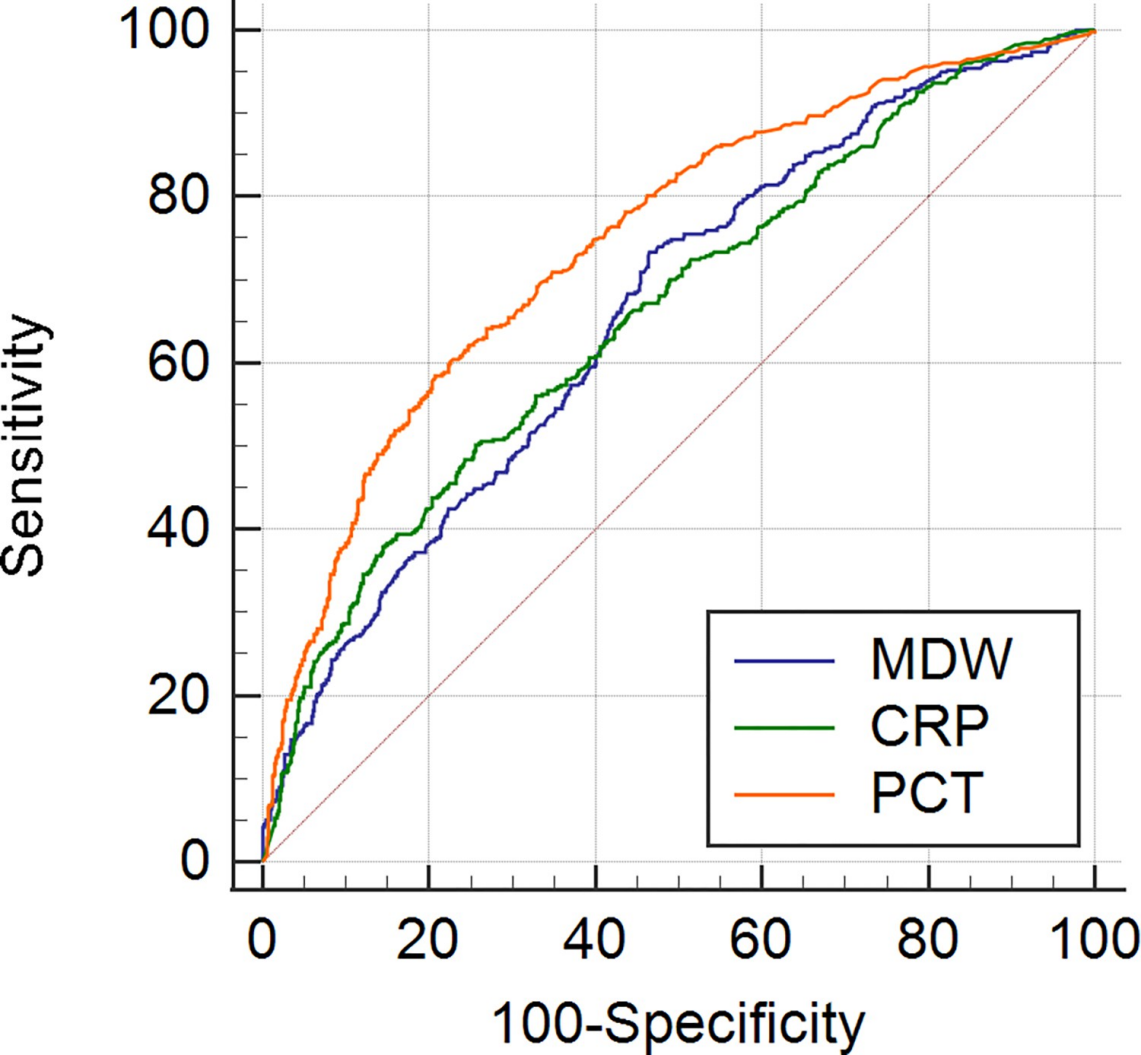
ROC curves of MDW, CRP and PCT for assessment of sepsis. ROC: receiver operating characteristics; MDW: monocyte distribution width; CRP: C-reactive protein; PCT: procalcitonin.

Five-hundred twenty patients were fulfilled Sepsis-3 criteria. For the detection of sepsis, MDW had a cut-off value of 21.7 with 74.0% sensitivity and 54.0% specificity, and AUC of 0.67 (95% CI, 0.64 to 0.69). The sensitivity, specificity, and AUC of CRP and PCT for the detection of sepsis are shown in Fig 1 and Table 2.

**Table 2:** AUC, sensitivity, and specificity of MDW, CRP and PCT for the assessment of sepsis.

Parameters (cut-off)	AUC median (IQR)	Sensitivity (%)	Specificity (%)
**MDW (21.71)**	**0.67 (0.64–0.69)**	**74.0**	**54.0**
CRP (11.16 mg/dL)	0.66 (0.63–0.68)	48.9	75.3
PCT (0.541 ng/mL)	0.75 (0.72–0.77)	58.3	79.3

AUC: Area under the curve; IQR: Inter quartile range; CRP: C-reactive protein; PCT: Procalcitonin; MDW: Monocyte distribution width.

Among the 1,404 patients, 191 patients had blood culture-positive (13.6%), 69 patients had blood culture-contamination (4.9%), and 1,144 patients had blood culture-negative (81.5%). The mean MDW, CRP, and PCT values of them are presented in Table 3.

**Table 3:** Comparison of MDW, CRP and PCT based on blood culture results.

Parameters	Number of patients Total (N/C/P) ^+^	Three groups by blood culture results			P-value
		Negative median (IQR)	Contamination median (IQR)	Positive median (IQR)	
**MDW** *	**1,404 (1,144/69/191)**	**21.8 (19.5–25.0)**	**22.3 (20.4–25.6)**	**27.0 (23.6–32.2)**	**<0.001**
Control	529 (495/34/0)	21.7 (18.6–24.2)	21.9 (19.8–24.9)	-	0.112
Infection	355 (298/10/47)	22.5 (19.8–25.1)	22.5 (21.4–25.0)	25.5 (21.4–27.7)	<0.001 (P vs. N) ** 0.193 (P vs. C) ^++^
Sepsis	520 (351/25/144)	23.2 (20.8–26.1)	22.6 (20.6–28.8)	27.8 (23.8–33.6)	<0.001 (P vs. N) 0.002 (P vs. C)
CRP, mg/dL	1,404 (1,144/69/191)	5.5 (1.1–12.7)	4.5 (1.5–15.1)	13.4 (5.7–21.3)	<0.001 (P vs. N) <0.001 (P vs. C)
PCT, ng/mL	1,221 (985/65/171)	0.17 (0.05–0.78)	0.15 (0.07–1.2)	2.8 (0.34–18.4)	<0.001 (P vs. N) <0.001 (P vs. C)

P-values were determined using a Mann Whitney U test.

MDW: monocyte distribution width; IQR: inter quartile range; CRP: C-reactive protein; PCT: procalcitonin.

*MDW results are divided three groups by Sepsis-3 criteria (control, infection, and sepsis groups).

^+^ N/C/P: the patient numbers in the groups of negative / contamination / positive by blood culture results.

**P vs. N: Statistics between positive group and negative group by blood culture results.

^++^P vs. C: Statistics between positive group and contamination group by blood culture results.

MDW results were significantly higher for the blood culture-positive group than for the blood culture-contamination group (P<0.001, 95% CI = -5.9 to -3.0) and blood culture-negative group (P<0.001, 95% CI = -5.8 to -4.2). Especially for the control group, MDW was not significantly different between the patients with blood culture-contamination and those with blood culture-negative (P = 0.112, 95% CI = -2.3 to 0.23). Among the infection group, MDW was statistically significant between patients with blood culture-positive and those with blood culture-negative (P<0.001 95% CI = 1.1 to 4.0), but there was no difference between those with blood culture-positive and blood culture-contamination (P = 0.193 95% CI = -1.3 to 5.5). Among the sepsis group, MDW was significantly higher for those with blood culture-positive than those with blood culture-negative (P<0.001, 95% CI = 3.4 to 5.6) and with blood culture-contamination (P = 0.002, 95% CI 1.7 to 7.0) groups.

For the comparison of MDW according to infection sites (Table 4), MDW was significantly higher for the patients with blood culture-positive than for others except the viral infections. In the infection and sepsis groups, MDW were significantly higher for the blood culture-positive than for others except the viral infection and co-infection, respectively.

**Table 4:** Comparison of MDW in the two groups (infection and sepsis) based on Sepsis-3 criteria according to blood culture results and their infection sites.

Blood culture results (Number of patients)	Infection sites	Number of patients (Infection group / Sepsis group)	Total MDW median (IQR)	MDW of two groups by Sepsis-3 criteria	
				Infection median (IQR)	Sepsis median (IQR)
**Positive** (N = 191)	**Blood**	**191 (47/144)**	**27.0 (23.6–32.3)**	**25.5 (21.4–27.7)**	**27.8 (23.8–33.7)**
	Blood only	97 (23/74)	25.8 (22.2–31.2)	24.7 (19.3–27.3)	26.4 (23.6–34.4)
	Blood with other sites	94 (24/70)	27.9 (24.0–32.4)	25.7 (22.3–31.7)	29.2 (24.3–33.1)
Negative (N = 684)	Respiratory tract	203 (74/129)	22.7 (20.1–25.1)	21.7 (19.3–25.1)	22.7 (20.5–25.1)
	Urinary tract	151 (83/68)	23.3 (20.2–25.4)	22.6 (19.6–25.0)	23.9 (21.0–26.3)
	Gastrointestinal tract	169 (91/78)	23.6 (20.7–25.9)	23.0 (20.1–25.5)	23.9 (21.0–26.5)
	Musculoskeletal system	35 (24/11)	22.3 (20.0–25.3)	21.2 (19.1–24.0)	25.3 (21.8–26.8)
	Others	26 (13/13)	23.0 (19.8–29.1)	20.3 (18.4–24.1)	25.3 (22.7–29.9)
	Co-infection*	73 (13/60)	22.7 (20.3–25.2)	23.7 (22.5–25.3)	22.3 (20.2–25.3)
	Viruses	27 (10/17)	25.7 (20.9–30.1)	23.5 (20.8–28.7)	26.1 (21.3–30.5)
	SARS-CoV-2	10 (4/6)	23.5 (20.9–30.4)	23.5 (21.5–29.5)	24.4 (20.4–30.6)
	Influenza	7 (3/4)	27.6 (20.4–28.4)	27.6 (20.4–27.8)	25.1 (19.7–28.8)
	Other viruses	10 (3/7)	28.1 (21.0–32.8)	21.4 (19.7–34.2)	30.0 (21.8–32.4)

*Co-infection is defined as a case of infection two or more sites with blood culture-negative.

MDW: Monocyte distribution width; IQR: Inter quartile range; N: Number of patients.

In the blood culture-positive only, MDW showed a significant difference between the infection and sepsis groups (P = 0.008, 95% CI = 0.98 to 7.2), but no difference was found in the patients with blood culture-positive concomitant other site infections. Viral infection showed a significant difference between the respiratory tract, urinary tract, musculoskeletal and co-infection group. MDW of 10 patients with SARS-CoV-2 was 23.5 (median, IQR 20.9–30.4) and 27.6 (median, IQR 20.4–28.4) for 7 patients with the influenza virus. MDW was 28.1 (median, IQR 21.0–32.8) for 10 patients with other viral infections (four hepatitis A virus (HAV) cases, one hepatitis C virus (HCV) case, one human-immunodeficiency virus (HIV) case, two varicella-zoster virus (VZV) cases, one cytomegalovirus (CMV) case and, one hantavirus case).

## Discussion

Early diagnosis is critical in providing appropriate management to patients with an infectious disease such as sepsis and bacteremia. While a blood culture test allows the causal bacteria to be identified and susceptibility of these bacteria to antibiotics to be analyzed, its interpretation is often challenging owing to false negative results and contaminating bacteria [23,24,27]. The purpose of this study was to identify the relationship between the blood culture results and MDW and to explore the usefulness of MDW as an early indicator of sepsis in the patients with a suspected infection. In this study, MDW was found to have high sensitivity in evaluating infection and sepsis, and to help interpret blood culture results. This study was conducted on a group of patients who performed a blood culture test to evaluate the relationship between MDW value and blood culture test results. Since all the patients included in this study were suspected of being infected, the term suspected infection group seems more appropriate than the term control group among the Sepsis-3 criteria. Therefore, the medical records of the patients were thoroughly investigated to exclude infection. To evaluate correctly infection status, clinicians used all available medical records including, emergency department admission, progression/discharge records, laboratory test, and radiological examination results during the hospitalization period. In this process, the usefulness was confirmed as an indicator of MDW’s Sepsis-3 assessment, and the results of the blood culture test and the relationship between infection sites were also evaluated. The patients analyzed in this study consisted only of patients who were tested for blood culture by clinicians’ request. They were already suspected infection clinically, and as a result, high prevalence of sepsis and high level of MDW were observed. For this reason, the MDW is higher in this study compared to other studies.

The sensitivity of MDW in this study was higher than that of CRP and PCT (Table 2), nevertheless this result indicated lower performance in comparison to previous studies [15,16,28]. There were some reasons for that. The first might be the fact that the patients in this study were those that were admitted to the emergency department due to a suspected infection and who subsequently underwent blood culture and other laboratory tests to identify the infection. The second might be there still remained a possibility of undetected infection although no evidence of infection could be found from the patients in the control group, therefore, the cut-off level of MDW for the performance assessment was 21.71 that was higher than that of previous study. MDW revealed high sensitivity and low specificity in contrast with CRP and PCT those were low sensitivity and high specificity. It means that MDW is a useful screening indicator for sepsis in the patients with a suspected infection, and it is effective test to confirm sepsis along with CRP and PCT results. The AUC of MDW for sepsis assessment in this study was lower than that of PCT (0.67 vs. 0.75) because the increase in PCT is specific to bacterial infection and the nonspecific increase in MDW is observed in various infectious diseases [3].

The level of MDW is higher in the blood collected using a tri-potassium (K_3_) EDTA tube than in the blood collected using a di-potassium (K_2_) EDTA tube, the previously reported MDW in the control group was 18.7 (median, IQR 16.6–23.5) with the K_2_ EDTA tube [16] and 19.0 (median, IQR 17.8–20.4) with the K_3_ EDTA tube [29]. In our hospital, we set up MDW normal reference value with 106 normal controls that was 17.2 (median, IQR 16.3–18.5) using K_3_ EDTA blood samples.

The usefulness of MDW as a predictor of sepsis is well presented in previous studies. Unlike other studies, this study confirmed that MDW may be useful as discriminating false positive blood cultures. It is certain that the increased MDW is related to blood culture positivity, which can be helpful in interpreting the blood culture results. This is supported by the fact that the difference of MDW was not significant between blood culture-contamination and blood culture-negative cases in the control group, in contrast to MDW was significantly different between the blood culture-contamination and blood culture-positive cases in the sepsis group (Table 3). In addition, the infection with blood culture-positive was definitely contributed to the increase in MDW (Table 4). This suggests a possible clinical application of MDW in determining whether the blood culture-contamination is due to true contaminants or pathogens. In some cases, even the contaminating bacteria of blood culture, it means the bacteria is not clinically important or normal flora, are regarded as pathogens [27]. The blood culture test should be performed within 12 hours of the admission for the percentage of blood culture positivity is higher. The low percentage of blood culture positivity should be taken into careful consideration in the interpretation of results, in that case, MDW can be a useful surrogate indicator.

The various sites of infection in the patients are categorized in Table 4, the contribution of blood infection in increasing MDW was certain. For COVID-19 infection, studies have reported an increase in MDW [18,19,20], and the variation of MDW can be used to monitor the disease in critical patients [30]. MDW was also reported to have increased in the influenza virus infection [18] and we found the same result in this study. In addition, the patients infected with HAV, HCV, HIV, VZV, CMV, or hantavirus were categorized into the viral infection group, and these also revealed an increased MDW. This implies that, similar to a positive blood culture, viral infections also have a role in the increase of MDW.

This study was conducted on patients admitted to the emergency department especially due to a suspected infection, who subsequently underwent a blood culture and other laboratory tests. The results revealed that blood culture-positive was the main contributing factor in the increase of MDW, and this could be applied in the interpretation of blood culture results for discriminating false positive blood cultures. Furthermore, MDW as a screening indicator is effective for assessment of sepsis in conjunction with CRP and PCT as confirmatory indicators, and MDW is especially useful for sepsis assessment in patients with a suspected infection.
